# Supplementary material for: Surface chemistry-mediated modulation of adsorbed albumin folding state specifies nanocarrier clearance by distinct macrophage subsets
Source: Nat Commun. 2021 Jan 28;12:648. doi: 10.1038/s41467-020-20886-7 (PMC7844416; doi:10.1038/s41467-020-20886-7)
Supplement: Supplementary file 3 — Reporting Summary [file 41467_2020_20886_MOESM3_ESM.pdf]

nature research

Corresponding author(s): Dr. Evan A. Scott, PhD.

Last updated by author(s): Nov 21, 2020

Reporting Summary

Nature Research wishes to improve the reproducibility of the work that we publish. This form provides structure for consistency and transparency in reporting. For further information on Nature Research policies, see our Editorial Policies and the Editorial Policy Checklist.

Statistics

For all statistical analyses, confirm that the following items are present in the figure legend, table legend, main text, or Methods section.

n/a	Confirmed	
[ ]	[x]	The exact sample size (n) for each experimental group/condition, given as a discrete number and unit of measurement
[ ]	[x]	A statement on whether measurements were taken from distinct samples or whether the same sample was measured repeatedly
[ ]	[x]	The statistical test(s) used AND whether they are one- or two-sided<br>*Only common tests should be described solely by name; describe more complex techniques in the Methods section.*
[x]	[ ]	A description of all covariates tested
[ ]	[x]	A description of any assumptions or corrections, such as tests of normality and adjustment for multiple comparisons
[ ]	[x]	A full description of the statistical parameters including central tendency (e.g. means) or other basic estimates (e.g. regression coefficient) AND variation (e.g. standard deviation) or associated estimates of uncertainty (e.g. confidence intervals)
[ ]	[x]	For null hypothesis testing, the test statistic (e.g. F, t, r) with confidence intervals, effect sizes, degrees of freedom and P value noted<br>*Give P values as exact values whenever suitable.*
[x]	[ ]	For Bayesian analysis, information on the choice of priors and Markov chain Monte Carlo settings
[x]	[ ]	For hierarchical and complex designs, identification of the appropriate level for tests and full reporting of outcomes
[ ]	[x]	Estimates of effect sizes (e.g. Cohen's d, Pearson's r), indicating how they were calculated

Our web collection on statistics for biologists contains articles on many of the points above.

Software and code

Policy information about availability of computer code

Data collection	No custom software was used for data collection in this manuscript.
Data analysis	A custom python (version 2.7.12) script was written to perform pairwise structural alignments presented in the supplementary materials of the manuscript. The backend of this software uses the structural alignment tool by UCSF Chimera to perform pairwise superposition of published protein structures obtained from the Protein Data Bank (PDB) and RMSD calculations. The PDB ID of each structure used in these structural alignments is provided in the manuscript. A summary of this custom software is provided in the manuscript. Furthermore, the python script is available upon request. A Code Availability statement has been provided in the manuscript.<br><br>Elsewhere in the manuscript, all commercial software that was used for data analysis is described. We have provided a short list here:<br>- Cytobank software suite (online software; version = Cytobank Community)<br>- Living Image software version 4.5.5. (PerkinElmer)<br>- PRIMUS (version 2.8.3.)<br>- Prism software (version 8.4.1; GraphPad Software)<br>- SasView (version 5.0)

For manuscripts utilizing custom algorithms or software that are central to the research but not yet described in published literature, software must be made available to editors and reviewers. We strongly encourage code deposition in a community repository (e.g. GitHub). See the Nature Research guidelines for submitting code & software for further information.

Data

Policy information about availability of data

All manuscripts must include a data availability statement. This statement should provide the following information, where applicable:

- Accession codes, unique identifiers, or web links for publicly available datasets
- A list of figures that have associated raw data
- A description of any restrictions on data availability

All relevant data are available from the authors upon request. A Data Availability statement can be found in the manuscript.

Field-specific reporting

Please select the one below that is the best fit for your research. If you are not sure, read the appropriate sections before making your selection.

[x] Life sciences [ ] Behavioural & social sciences [ ] Ecological, evolutionary & environmental sciences

For a reference copy of the document with all sections, see nature.com/documents/nr-reporting-summary-flat.pdf

Life sciences study design

All studies must disclose on these points even when the disclosure is negative.

Sample size	The selection of a minimum of 5 mice per group was calculated from a two tailed power analysis with a confidence level of 95% using standard freely available software G Power 3.1.9.2, as recommended by the literature (Charan et al.,"How to calculate sample size in animal studies", J Pharmacol Pharmacother. 2013 OctDec; 4(4): 303–306). Additionally, we have previously found that this sample size allows statistically significant differences to be found for biodistribution and immunological studies using PEG-b-PPS nanomaterials (Velluto et al., Biomaterials, Volume 32, Issue 36, December 2011, Pages 98399847; Stano et al. Volume 34, Issue 17, June 2013, Pages 4339–4346). For in vitro and materials characterization, sample sizes were chosen to be sufficiently large to allow the examination of statistically significant differences based on previous publications or experiments using similar characterization methods.
Data exclusions	No data were excluded from the analyses.
Replication	Biodistribution analyses were performed with a sample size calculated as described above. Aside from these studies, experiments were replicated using at least two independent experiments.
Randomization	Mice were allocated randomly into treatment groups. Randomization is only applicable to the mouse studies in the present work, and is not relevant to the other experiments presented in this work.
Blinding	For mouse studies, nanoparticle treatment group blinding was employed at the time of data collection and analysis. Aside from the mouse studies, blinding is not relevant to the other experiments presented in this work.

Reporting for specific materials, systems and methods

We require information from authors about some types of materials, experimental systems and methods used in many studies. Here, indicate whether each material, system or method listed is relevant to your study. If you are not sure if a list item applies to your research, read the appropriate section before selecting a response.

Materials & experimental systems

n/a	Involved in the study	
[ ]	[x]	Antibodies
[ ]	[x]	Eukaryotic cell lines
[x]	[ ]	Palaeontology and archaeology
[ ]	[x]	Animals and other organisms
[x]	[ ]	Human research participants
[x]	[ ]	Clinical data
[x]	[ ]	Dual use research of concern

Methods

n/a	Involved in the study	
[x]	[ ]	ChIP-seq
[ ]	[x]	Flow cytometry
[x]	[ ]	MRI-based neuroimaging

Antibodies

Antibodies used	Antibodies used for in vivo studies:<br>Alexa Fluor 700 Ly-6C anti-mouse (BioLegend; clone HK1.4; Cat #: 128024; Lot #: B297755)<br>Alexa Fluor 700 Ly-6G anti-mouse (BioLegend; clone 1A8; Cat #: 127622; Lot #: B266371)<br>APC anti-mouse MHCII (I-A/I-E) (BioLegend; clone M5/114.15.2; Cat #: 107614; Lot #: B287194)<br>APC CD169 anti-mouse (BioLegend; clone 3D6.112; Cat #: 142418; Lot #: B279352)

Brilliant Violet 510 CD3 anti-mouse (BioLegend; clone 145-2C11; Cat #: 100353; Lot #: B292882)
Brilliant Violet 510 anti-mouse CD19 (BioLegend; clone 6D5; Cat #: 115545; Lot #: B279187)
Brilliant Violet CD24 anti-mouse (BioLegend; clone M1/69; Cat #: 101831; Lot #: B281776)
Brilliant Violet 510 anti-mouse NK-1.1 (BioLegend; clone PK136; Cat #: 108737; Lot #: B262007)
BUV395 rat anti-mouse CD45 (BD Horizon; clone 30-F11; Cat #: 564279; Lot #: 9177420)
PE Anti-CD11c (BioLegend; clone N418; Cat #: 117308; Lot #: B278350)
PE/Dazzle 594 anti-mouse F4/80 (BioLegend; clone BM8; Cat #: 123146; Lot #: B288537)
PE/Dazzle 594 CD64 anti-mouse (BioLegend; clone X54-5/7.1; Cat #: 139320; Lot #: B272823)
PerCP/Cyanine5.5 anti-mouse/human CD11b (BioLegend; clone M1/70; Cat #: 101228; Lot #: B247471)
Purified anti-mouse CD16/32 (BioLegend; clone 92; Cat #: 101302; Lot #: B282595)

Antibodies used for in vitro studies:
FITC Rat anti-mouse CD204 (Bio-RAD; clone 2F8; Cat #: MCA1322FA; Lot #: 1706)

Validation

Validation statements can be found on the manufacturer's website:
https://www.biolegend.com/
https://www.bdbiosciences.com/
https://www.bio-rad-antibodies.com/

In this validation section, we have provided information for each antibody from the manufacturer website (this information is obtained from the website links listed above, and the details of each specific product is provided in the "Antibodies used" section of this document). The descriptions below are paraphrased summaries from these websites and are provided for the purpose of this document. If more information is desired, all information necessary to look up additional information regarding the antibody validation performed by the manufacturer is provided elsewhere in this document (see manufacturer information, clone information, catalog numbers and lot numbers for each antibody in the "Antibodies used" section in this document, which can be used to access more product information on the manufacturer website).

Validation description for antibodies used for in vivo studies:
Alexa Fluor 700 Ly-6C anti-mouse (BioLegend) - validated by flow cytometry on C57BL/6 bone marrow cells.
Alexa Fluor 700 Ly-6G anti-mouse (BioLegend) - validated by flow cytometry on C57BL/6 bone marrow cells.
APC anti-mouse MHCII (I-A/I-E) (BioLegend) - validated by flow cytometry on C57BL/6 mouse splenocytes stained with anti-I-A/I-E or or rat IgG2b, κ isotype control.
APC CD169 anti-mouse (BioLegend) - validated by flow cytometry on C57BL/6 mouse bone marrow cells that were stained with Ly-6G, CD169 (clone 3D6.112), or rat IgG2a, κ.
Brilliant Violet 510 CD3 anti-mouse (BioLegend) - validated by flow cytometry on C57BL/6 mouse splenocytes stained with anti-CD3e or Armenian hamster IgG isotype control.
Brilliant Violet 510 anti-mouse CD19 (BioLegend) - validated by flow cytometry on C57BL/6 mouse splenocytes stained with anti-CD19 or rat IgG2a, κ control.
Brilliant Violet CD24 anti-mouse (BioLegend) - validated by flow cytometry on C57BL/6 mouse splenocytes stained with anti-CD24 or IgG2b isotype control.
Brilliant Violet 510 anti-mouse NK-1.1 (BioLegend) - validated by flow cytometry on C57BL/6 mouse splenocytes stained with anti-CD49b/DX5 and anti-NK1.1 or mouse IgG2a, κ isotype control.
BUV395 rat anti-mouse CD45 (BD Horizon) - validated by flow cytometry on mouse splenic leukocytes pre-incubated with purified rat anti-mouse CD16/CD32 antibody and stained with anti-CD45 or rat IgG2b, κ isotype control.
PE Anti-CD11c (BioLegend) - validated by flow cytometry on C57BL/6 mouse splenocytes stained with anti-I-A/I-E, anti-PE N418, or Armenian hamster IgG isotype control.
PE/Dazzle 594 anti-mouse F4/80 (BioLegend) - validated by flow cytometry on thioglycolate-elicited BALB/c mouse peritoneal macrophages stained with anti-F4/80 (clone BM8), or rat IgG2a, κ isotype control.
PE/Dazzle 594 CD64 anti-mouse (BioLegend) - validated by flow cytometry on C57BL/6 mouse bone marrow cells stained with anti-CD11b (clone M1/70) and anti-CD64 (clone X54-5/7.1) or mouse IgG1, κ isotype control.
PerCP/Cyanine5.5 anti-mouse/human CD11b (BioLegend) - validated by flow cytometry on C57BL/6 mouse splenocytes were blocked with TruStain FcX™ (anti-mouse CD16/32) antibody, then were stained with anti-Ly-6G/Ly-6C (Gr-1) and anti-CD11b or rat IgG2b, κ isotype control.
Purified anti-mouse CD16/32 (BioLegend) - validated by flow cytometry on C57BL/6 mouse splenocytes that were stained with purified 93, followed by anti-rat IgGs.

Antibodies used for in vitro studies:
FITC Rat anti-mouse CD204 (Bio-RAD) - validated by flow cytometry by staining J774.2 cells with anti-CD11b and either anti-CD204 or anti-Rat IgG2b isotype control.

Eukaryotic cell lines

Policy information about cell lines

Cell line source(s)	Murine RAW 264.7 macrophages were purchased from ATCC.

Authentication	Cell authentication statements can be found on the manufacturer website: https://www.atcc.org/<br><br>ATCC states on their website that their company uses morphology, karyotyping, and PCR based approaches in their cell line authentication procedures.
Mycoplasma contamination	All cell lines were confirmed to not contain mycoplasma contamination.
Commonly misidentified lines (See ICLAC register)	To our knowledge, none of the lines used in this study are commonly misidentified.

Animals and other organisms

Policy information about studies involving animals; ARRIVE guidelines recommended for reporting animal research

Laboratory animals	8-to-12 week old C57BL/6J mice were purchased from The Jackson Laboratory. Female mice (90 day old) were used in the mouse studies. All mice were housed and maintained in the Center for Comparative Medicine at Northwestern University at 18-23°C with 40-60% humidity, and 12h/12h dark/light cycle.
Wild animals	This study did not involve wild animals.
Field-collected samples	This study did not involve field-collected samples.
Ethics oversight	All experiments conducted in vivo were performed in accordance with animal protocols approved by the Institutional Animal Care and Use Committee (IACUC) at Northwestern University.

Note that full information on the approval of the study protocol must also be provided in the manuscript.

Flow Cytometry

Plots

Confirm that:

☒ The axis labels state the marker and fluorochrome used (e.g. CD4-FITC).

☒ The axis scales are clearly visible. Include numbers along axes only for bottom left plot of group (a 'group' is an analysis of identical markers).

☒ All plots are contour plots with outliers or pseudocolor plots.

☒ A numerical value for number of cells or percentage (with statistics) is provided.

Methodology

Sample preparation	Details regarding all sample preparation procedures are listed in the manuscript.<br><br>Sample preparation procedures used for biodistribution analyses: (these descriptions are taken directly from the methods section of our manuscript and describe sample preparation in detail)<br>- Organ harvesting: Mice were euthanized four hours after administration. Whole blood was collected in heparin-treated tubes by cardiac puncture. The spleen (SP), liver (LV), lymph nodes (LN), kidneys (K), lungs (LG), and heart (H) was dissected after performing a whole-body PBS perfusion. A total of six lymph nodes were collected per mouse (2 axial, 2 brachial and 2 inguinal).<br>- Preparation of single cell suspensions: Single cell suspensions were prepared from dissected spleen, liver, lymph nodes, kidneys, and lungs. For all cellular staining with antibodies, cells were washed with PBS. FcR binding was subsequently blocked by incubating cells with anti-mouse CD16/32 (Biolegend) and cells were simultaneously stained with fixable Zombie Aqua (used for in vitro experiments) or fixable Zombie Violet (used for in vivo experiments) viability dye (Biolegend) for 20 min at 4C to distinguish live cells from dead cells. Afterwards, cells were stained using cocktails of fluorophore-conjugated antibodies.
Instrument	Flow cytometry was performed using a BD LSRFortessa 6-Laser Flow Cytometer (16 color compatible instrument) maintained by the Robert H. Lurie Comprehensive Cancer Center (RHLCCC) Flow Cytometry Facility at Northwestern University.
Software	The cytobank analysis suite was used to analyze the acquired flow cytometry data.
Cell population abundance	This manuscript used flow cytometry, however, we did not perform physical cell sorting (i.e. FACS) in any of the experiments.
Gating strategy	The gating strategy is described in detail in both the methods section and the supplementary materials of the manuscript.

☒ Tick this box to confirm that a figure exemplifying the gating strategy is provided in the Supplementary Information.
